# Perforation of the Interventricular Septum by a Temporary Transvenous Pacing Lead Detected by Cardiac Point of Care Ultrasound (POCUS)

**DOI:** 10.24908/pocusj.v10i01.18271

**Published:** 2025-04-15

**Authors:** Pablo Blanco, Liliana Figueroa

**Affiliations:** 1 Intermediate Care Unit, Hospital “Dr. Emilio Ferreyra”, Necochea, ARG; 2 Department of Teaching and Research, Hospital “Dr. Emilio Ferreyra”, Necochea, ARG

**Keywords:** bedside ultrasonography, articial cardiac pacemaker, ultrasound guided procedures

## Abstract

Several complications can arise during or shortly after the placement of a temporary transvenous pacemaker (TTP), some of which can be potentially devastating. A TTP was successfully placed under the guidance of point of care ultrasound (POCUS) and intracavitary electrocardiogram (ECG) in a middle-aged woman with symptomatic complete atrioventricular block. Three days post-insertion, pacemaker malfunction was observed along with the migration of the pacing lead to the left ventricular apex on cardiac POCUS. The lead was promptly repositioned, and no complications were noted on subsequent cardiac POCUS examinations. To prevent complications and aid early detection of complications associated with TTPs, cardiac POCUS should be strongly considered.

## Introduction

Temporary transvenous pacing is primarily indicated as a life-saving intervention in symptomatic bradyarrhythmias and can also be valuable in managing incessant ventricular tachycardia through overdrive pacing [1]. The procedure, however, carries inherent risks, including complications related to venous access (e.g., bleeding, pneumothorax), device malfunction (e.g., failure to capture or sense), and pacing lead complications, such as cardiac perforation and pericardial tamponade during or shortly after insertion [2]. Although rare, interventricular septal perforation (ISP) with lead migration into the left ventricle (LV) has been documented [3]. We present a case of ISP confirmed by cardiac point of care ultrasound (POCUS), which also guided the successful repositioning of the pacing lead. Additionally, we briefly review the role of POCUS in guiding temporary transvenous pacemaker (TTP) placement and follow-up monitoring.

## Case Presentation

**Figure 1.**
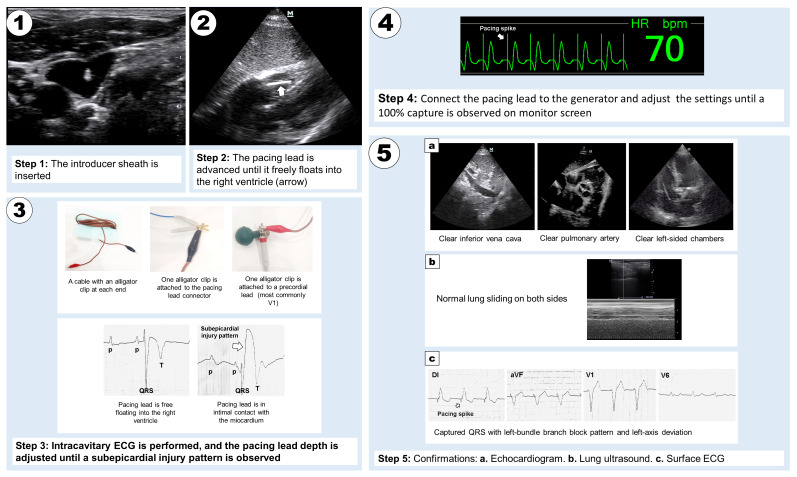
Steps for performing temporary transvenous pacing using point of care ultrasound (POCUS) and intracavitary electrocardiogram (ECG) guidance.

A 63-year-old woman was admitted with symptomatic complete atrioventricular block. Her medical history was notable for arterial hypertension, managed with enalapril. A TTP was successfully placed via the right internal jugular vein under the guidance of POCUS and intracavitary electrocardiogram (ECG), following this procedure [1]: A 7F introducer sheath was inserted into the right internal jugular vein under real-time ultrasound visualization. Subsequently, a 6F, torque-directed, 110 cm temporary pacing catheter was advanced. A second clinician monitored its progression into the right ventricle via subcostal or apical four-chamber cardiac POCUS views. Intracavitary electrocardiography was performed once the pacing lead was floating in the right ventricle. This involved connecting the distal pole of the pacing catheter to a precordial ECG lead using alligator clips, with the limb leads attached in the usual manner. Adjustments to the lead depth were made until a subepicardial injury pattern was identified on lead V1, confirming intimal contact between the pacing lead and the right ventricular wall. The pacemaker generator was then connected to the pacing lead connnector, and ventricular capture was optimized to ensure effective pacing, as confirmed on the monitor. The final verification of lead placement was achieved through cardiac POCUS imaging, which confirmed proper positioning within the right ventricle. Acceptable lead locations included the apex, the interventricular septum—where the lead was ultimately positioned in our patient—the lateral wall, or the right ventricular outflow tract. Misplacement into the pulmonary artery, inferior vena cava, or left-sided cardiac chambers was definitively excluded. Additionally, lung ultrasound was performed to exclude pneumothorax, and surface ECG was evaluated to confirm proper pacing function. The latter demonstrated a paced rhythm characterized by the presence of pacing spikes followed by broad QRS complexes, displaying a left bundle branch block pattern with left-axis deviation (the complete procedure is illustrated in Figure 1).

**Figure 2.**
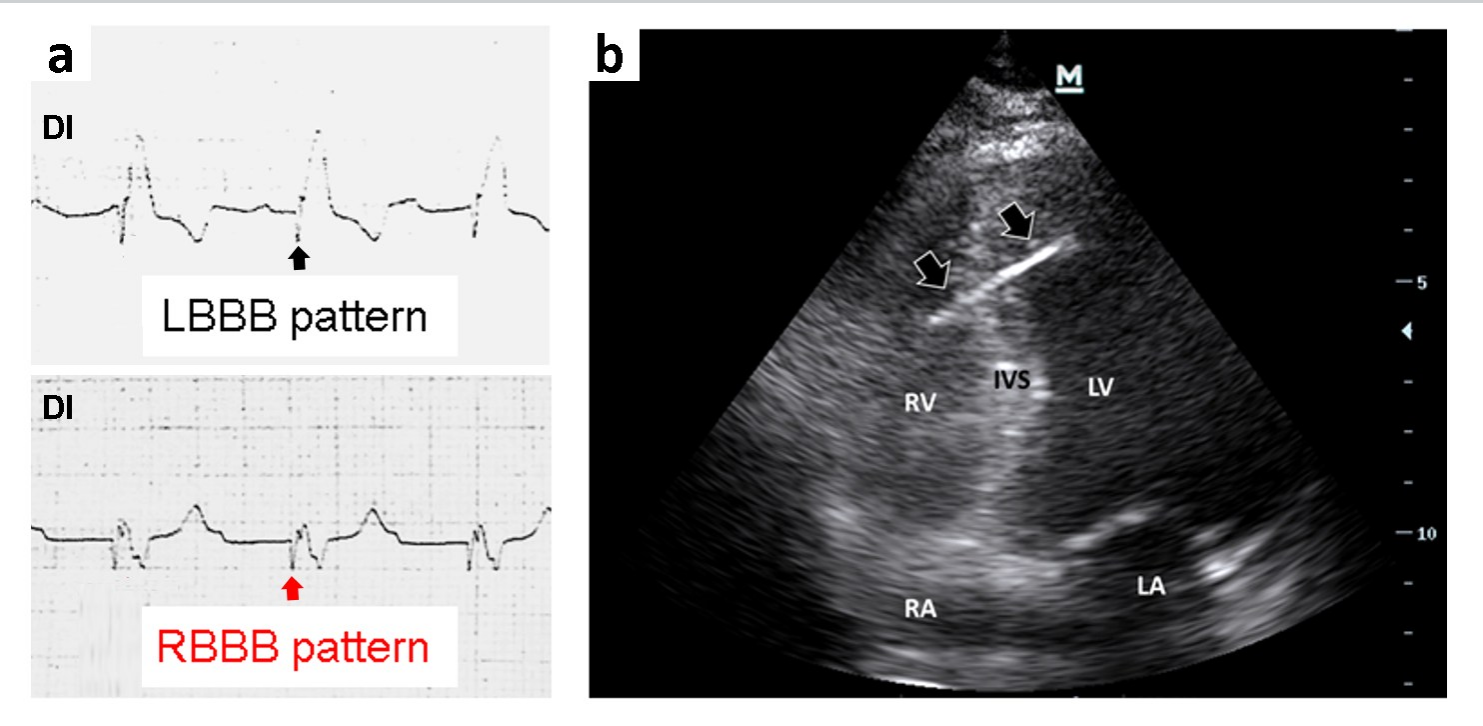
(a) Surface electrocardiogram (ECG) tracing (lead D1, shown for simplicity to avoid displaying the entire ECG) demonstrating alternating ventricular capture patterns, characterized by left bundle branch block (LBBB) and right bundle branch block (RBBB) morphologies. The black arrow indicates a pacemaker spike associated with right ventricular capture, while the red arrow highlights a pacemaker spike corresponding to left ventricular capture. (b) Pacing lead traversing from the right ventricle to the left ventricle following perforation of the apical interventricular septum. Arrows: pacing lead; LV: left ventricle; RV: right ventricle; RA: right atrium; LA: left atrium; IVS: interventricular septum.

On the third day post-admission, the patient reported a mild chest discomfort localized over the cardiac apex, and the ECG revealed erratic ventricular capture, characterized by alternating a left bundle branch block and right bundle branch block morphologies (Figure 2a). The patient's vital signs remained stable, and physical examination was unremarkable. Cardiac POCUS identified that the pacing lead had perforated the apical interventricular septum and extended into the left ventricular apex (Figure 2b and Video 1). The lead was promptly repositioned into the right ventricle under cardiac POCUS and intracavitary ECG guidance. Subsequent serial cardiac POCUS exams demonstrated no evidence of an interventricular septal defect on two-dimensional or color Doppler imaging and no pericardial effusion. Given the low likelihood of right-to-left shunting, an agitated saline test was not performed.

## Discussion

Lead-related complications associated with TTP placement can occur during or shortly after the procedure. Cardiac perforation and subsequent pericardial tamponade, which occur at a rate of 0.6%, represent the most feared complications as they carry a fivefold increased risk of mortality [2]. Another rare but significant complication is ISP with left ventricular pacing, which is associated with thrombus formation and cardioembolic events [3,4]. The exact incidence of ISP, however, remains undocumented to date. ISP may arise from factors such as lead stiffness, excessive insertion force or over-manipulation, septal lead positioning (as observed in our patient) and the use of blind placements [3–5]. In some patients with anatomically soft or “cheesy” septal tissue, excessive lead movement may predispose the septum to erosion or perforation [5]. In our case, we hypothesize that progressive erosion of the interventricular septum occurred over the course of a few days, ultimately leading to perforation and migration of the pacing lead into the LV.

TTP related complication can be diagnosed through pacemaker function assessment, chest radiography, fluoroscopy, computed tomography, or cardiac POCUS, with the latter being particularly valuable at the point of care.

To minimize adverse events during TTP placement, blind insertions should be avoided. Balloon-tipped catheters are also preferred over rigid ones, as the former are associated with faster insertions and lower complication rates [6]. Ultrasound-guided insertion combined with intracavitary ECG has been demonstrated to safely guide the pacing lead to the right ventricle, ensuring proper device sensing and capture [1]. Moreover, ultrasound guidance reduces the incidence of vascular access-related complications [7]. Serial cardiac POCUS monitoring is crucial for detecting anomalies that may develop even after an initially uncomplicated placement. When transthoracic windows are inadequate, point of care transesophageal echocardiography can provide additional diagnostic value [8].

## Conclusions

POCUS is a vital tool both for preventing complications associated with TTP and their early detection. Physicians placing TTP should consider incorporating POCUS into their practice.


